# Salivary Antioxidant Profile in Patients with Systemic Sclerosis and Periodontitis

**DOI:** 10.1155/2023/7886272

**Published:** 2023-04-24

**Authors:** Stefan I. Sredojevic, Slavica Pavlov Dolijanovic, Ivan Dozic, Jovana Kuzmanovic Pficer, Zoran Aleksic, Natasa S. Nikolic-Jakoba

**Affiliations:** ^1^Department of Periodontology, School of Dental Medicine, University of Belgrade, Dr Subotica 4, Belgrade, Serbia; ^2^Institute of Rheumatology, School of Medicine, University of Belgrade, Resavska 69, Belgrade, Serbia; ^3^General and Oral Biochemistry, School of Dental Medicine, University of Belgrade, Dr Subotica 4, Belgrade, Serbia; ^4^Biomedical Statistics and Informatics, School of Dental Medicine, University of Belgrade, Dr Subotica 4, Belgrade, Serbia

## Abstract

**Objective:**

The aim of the present study was to compare periodontal status and antioxidant profile in unstimulated saliva of systemic sclerosis (SSc) patients with periodontitis and systemically healthy periodontitis patients.

**Design:**

Twenty patients with established diagnoses of systemic sclerosis and periodontitis (SSc group) and 20 systemically healthy individuals with periodontitis (P group) were enrolled in the study. Clinical periodontal parameters (clinical attachment level (CAL), gingival recession (GR), periodontal probing depth (PPD), and gingival index (GI)) and concentration of uric acid (UA), superoxide dismutase (SOD), and glutathione peroxidase (GPX) in unstimulated saliva samples were assessed.

**Results:**

There were significantly higher mean values of CAL (4.8 ± 0.21 mm versus 3.18 ± 0.17 mm; *p* ≤ 0.001) and GR (1.66 ± 0.90 mm versus 0.46 ± 0.54 mm; *p* ≤ 0.001) in the SSc group when compared to the P group. Significantly higher level of GPX (*p* ≤ 0.001) and SOD (*p* ≤ 0.001) in unstimulated saliva was detected in the SSc group in comparison with the P group. The specific activity of UA did not significantly differ between the two groups (*p* = 0.083).

**Conclusion:**

The results may indicate higher periodontal destruction and antioxidant perturbations in unstimulated saliva of SSc patients with periodontitis compared to systemically healthy periodontitis patients.

## 1. Introduction

Systemic sclerosis (SSc) is a rare autoimune systemic disease of unknown origin that is characterized by tissue fibrosis and vasculopathy of skin and major internal organs [1]. According to the degree of skin involvement and prognosis, SSc is divided into two subsets—limited and diffuse cutaneous scleroderma [2]. The incidence of scleroderma varies from 0.6 to 2.4 per million per year in the adult population [3]. The most commonly affected organs include the skin, kidney, heart, and exocrine glands such as salivary glands [4]. Fibrotic changes in salivary glands in combination with medication can cause reduced saliva production and may have multiple implications on periodontal health [5, 6]. A recent systematic review demonstrated a positive correlation between systemic sclerosis and periodontal disease [7].

Periodontitis is a chronic multifactorial inflammatory disease associated with dysbiotic biofilm, characterized by progressive destruction of the tooth-supporting apparatus [8]. The most important pathogenic feature that scleroderma and periodontitis have in common is inflammatory-mediated tissue destruction that finally results in loss of function [9]. For both diseases, the presence of unneutralized free radicals is considered to be responsible for tissue destruction, either by direct reaction with cells or via the release of proinflammatory cytokines [10, 11].

Although the pathogenesis of systemic sclerosis and periodontitis has not been understood completely, there is unambiguous evidence that oxidative stress plays a vital role. The sign of oxidative stress arises when the production of reactive oxygen species (ROS) overcomes the capacity of the antioxidant system. The result of disturbed oxidative equilibrium in SSc includes vascular hyperreactivity, endothelial cell apoptosis, and impaired angiogenesis. The major source of ROS in scleroderma is ischemic reperfusion injury, fibroblasts and macrophage hyperactivation, and deregulated metabolism of NO free radicals [12].

Antioxidant system activity consists of enzymatic direct neutralization of ROS and nonenzymatic neutralization of secondary oxidative product. The main enzymatic scavenger of free radicals in the saliva is glutathione peroxidase (GPX), produced in salivary glands, while uric acid (UA) plays a dominant nonenzymatic antioxidant role [13]. Superoxide dismutase (SOD) occurs in a few isoenzymes in saliva and has a secondary antioxidative role [14].

Despite the function of oxidative stress in the pathogenesis of both scleroderma and periodontitis being well established, to our knowledge, there is no available study analyzing the antioxidant profile of unstimulated saliva in SSc patients with periodontitis.

The goal of the present study was to compare the antioxidant profile in unstimulated saliva of SSc patients with periodontitis and systemically healthy periodontitis patients.

## 2. Material and Methods

### 2.1. Study Population

The study was performed from February 2019 to April 2022 at the Department of Periodontology, School of Dental Medicine, University of Belgrade, Serbia, and the Institute for Rheumatology, Belgrade. Thirty-two patients with established diagnoses of systemic sclerosis (SSc) according to the American Rheumatism Association criteria for scleroderma [15] were referred for periodontal examination from the Institute for Rheumatology, Belgrade, to the Department of Periodontology, School of Dental Medicine, University of Belgrade, Serbia. Twenty systemic sclerosis patients with periodontitis who matched the inclusion criteria were enrolled in the present study (SSc group). Twelve patients with systemic sclerosis were not included in the study as they had less than 12 teeth (*n* = 10) or did not want to participate (*n* = 2). Twenty systemically healthy individuals with periodontitis were selected for the control group (P group) from patients who were seeking care for periodontal problems at the Department of Periodontology, School of Dental Medicine, University of Belgrade, Serbia.

At the screening appointment, all individuals received a detailed explanation about the present study, after which a complete oral examination had been conducted. The study protocol was approved by the institutional ethical committee (permission reference no. 29/1-47) in accordance with the Helsinki Declaration of 1975, as revised in 2000. The study was registered on ClinicalTrials.gov, ID number: NCT05215431.

Written informed consent was obtained from each patient following the explanation of the nature, purpose, and potential risks of the study.

Inclusion criteria for patients with systemic sclerosis and periodontitis (SSc group) and systemically healthy periodontitis patients (P group) included the following: (1) patients aged ≥18 years; (2) presence of at least 12 teeth; (3) stage II or III periodontitis; and (4) grade B periodontitis.

Criteria defining stage II periodontitis included the value of the clinical attachment level (CAL) interdental from 3 to 4 mm; X-ray detected loss of alveolar bone (15-33%) with maximum periodontal probing depth (PPD) up to 5 mm at the most pronounced periodontal destruction site. Stage III periodontitis was defined by CAL interdentally ≥5 mm; X-ray detected loss of more than a third of the alveolar bone, *PPD* ≥ 6 *mm* at the most pronounced periodontal destruction site with loss of up to four teeth due to periodontitis [16].

Since previous periodontal records of screened patients were not available, the bone level/age (BL/A) ratio was calculated from the panoramic X-ray. Grade B periodontitis was established if BL/A was between 0.25 and 1.0.

The exclusion criteria for both groups of patients included concurrent inflammatory-mediated rheumatic diseases, pregnancy or lactation, active cigarette smokers, a history of periodontal therapy, antibiotics, or nonsteroidal drug usage in the past 6 months.

### 2.2. Clinical Rheumatologic Assessment

A complete clinical rheumatologic assessment was conducted at the Institute for Rheumatology, Belgrade, Serbia. The skin involvement in systemic sclerosis patients was assessed using a modified Rodnan skin score (MRSS) [17], while disease activity was determined by an activity score according to the European scleroderma study group and research [18]. The duration of the disease was considered as the time since the first onset of the non-Reynaulds phenomenon up to the study visit, based on patient self-report. The differentiation between the limited and diffuse types of SSc was based on skin involvement, proximal or distal to elbows, and knees [2].

### 2.3. Saliva Collection and Preparation

Saliva samples were collected at the baseline visit directly before the clinical periodontal examination. All participants were scheduled between 8 and 10 A.M. and received instructions not to eat nor drink (except water) a minimum of two hours before the visit. The patients were instructed to sit upright and expectorate into a sterile plastic tube (Salivette, Sarstedt, Germany) immersed into crushed ice for 15 minutes. Immediately after volume measurement, sealed tubes with collected saliva were transported to the laboratory where they were centrifuged at 4000 rpm, 9 g for 20 min at 4°C. The free-of-cell supernatants were then frozen and kept at -80°C until analyzed.

### 2.4. Determination of Antioxidants in Saliva Samples

Antioxidants were determined using the colorimetric method and commercial kits for SOD activity (Ransod; Randox Laboratories Ltd., Crumlin, UK), GPX activity (Ransel; Randox Laboratories Ltd.), and uric acid (UA, uric acid liquicolor; HUMAN Gesellschaft fur Biochemica und Diagnostica mbH).

### 2.5. Clinical Oral Examination

Clinical assessment was performed by one calibrated examiner (SS) under standardized conditions at the Department of Periodontology, School of Dental Medicine, University of Belgrade. The assessment included estimation of dental and periodontal health, measurement of interincisal distance (IID), and evaluation of TMJ function. The interincisal distance was defined as the distance between the maxillary and mandibular central incisors at the midline when the mouth was maximally opened. Any presence of symptoms and signs of TMJ disorders such as crepitation, movement limitation, and pain was also noted.

#### 2.5.1. Intraoral Examination

After the number of present teeth was noted down, periodontal parameters such as periodontal probing depth (PPD), clinical attachment level (CAL), and gingival recession (GR) were recorded on six sites of each tooth using a manual periodontal probe (North Carolina–Hu-Friedy, Chicago, IL, USA). Periodontal probing depth (PPD) was determined as the measured distance from the gingival margin to the bottom of the periodontal pocket. The measured distance from the cementoenamel junction (CEJ) to the gingival margin was defined as a gingival recession (GR). Distance from the CEJ to the bottom of the periodontal pocket was defined as clinical attachment level (CAL) and calculated indirectly as the sum of PPD and GR values.

The Loe and Silness gingival index (LSGI) was used for rating the degree of gingival inflammation [19]. Evaluation of oral hygiene status was recorded with a Silness–Loe plaque index (PI). In addition, the decayed, missing, filled teeth index (DMFT) as described by Klein was used for dental status determination of each participants [20].

### 2.6. Statistical Analysis

All statistical analyses were done in SPSS program version 22.0 (SPSS Inc., Chicago, IL). Numerical data were presented as mean and standard error (SE). The Kolmogorov-Smirnov test was used for testing normal distribution. We used for comparison between two groups the independent *t*-test or the Mann–Whitney *U* test. Correlation between clinical parameters and antioxidant values was presented with the Pearson and Spearman correlation coefficients. Data were statistically significant when *p* value was set as 0.05. Participants were collected according to inclusion criteria. Based on the results of the study, the post hoc-achieved power was 82.98%. Power for 40 respondents was calculated for the difference between two independent means, *α* = 0.05, and effect size dz was calculated based on the mean and SD of the difference between two independent means for SOD. This was performed in G^∗^Power program version 3.1.9.4. (Germany).

## 3. Results

### 3.1. Patients' Characteristics

The study population included 20 individuals with systemic sclerosis and periodontitis (70% female) with a mean age of 56.67 (±1.96) and a median duration of systemic sclerosis of 5.22 (±0.62). The diffuse cutaneous scleroderma was more frequent than the limited form of the disease (80%). The clinical features, autoantibody status, and treatment of patients with SSc are summarised in Table 1. The control group was composed of 20 systemically healthy individuals with periodontitis (60% female) with a mean age of 58.45 (±2.32).

### 3.2. Dental and Periodontal Findings

The mean value of the DMFT index in the SSc group was significantly higher than for control subjects (*p* ≤ 0.001). Significantly higher mean CAL and GR were detected in the SSc group (*p* ≤ 0.001). However, there were no statistically significant differences for PPD (*p* = 0.123) and GI values (*p* = 0.107) between the groups. PI was significantly higher in the SSc group compared with the P group (*p* ≤ 0.001). All patients in the SSc group had stage III periodontitis, while 85% of cases in the P group had stage III periodontitis and the remaining 15% had stage II periodontitis. Patients in both study groups were diagnosed with grade B periodontitis. Dental and periodontal findings are listed in Table 2. Nonsignificant correlations were observed between clinical rheumatological parameters (disease duration, disease activity, and MRSS) and clinical periodontal variables (data not shown).

### 3.3. Specific Activity of Salivary Antioxidants

Comparison of salivary antioxidant values showed that the specific activity of GPX and SOD in unstimulated saliva was significantly elevated in the SSc group than in the P group (*p* ≤ 0.001 and *p* = 0.010, respectively). Specific activity of UA did not significantly differ between SSc and P groups (*p* = 0.083) (Table 2).

The Pearson analysis of correlations among salivary antioxidants and periodontal parameters in the SSc group is depicted in Table 3. No significant correlation between measured antioxidants and periodontal clinical parameters was established in both group (Table 4). A higher level of GPX was positively correlated with a higher disease activity score, while the higher level of SOD positively correlated with higher MRSS (Table 5).

## 4. Discussion

The present study compared the salivary antioxidant profile of patients with systemic sclerosis and periodontitis with systemically healthy periodontitis patients. Results of the study revealed increased specific activity of GPX and SOD in unstimulated saliva in the SSc patients, while there were no differences in UA specific activity between the groups.

In a recent systematic review conducted by Doridot et al., ROS were marked as crucial players in the pathophysiology of systemic sclerosis [21]. They have a major role in endothelial cell apoptosis and the differentiation of fibroblasts to myofibroblasts leading to vascular impairment and tissue fibrosis [22, 23]. On the other hand, several studies revealed that an imbalance between ROS and antioxidants in saliva may be responsible for the progression of oral cavity-associated diseases [24, 25]. An imbalance in the salivary antioxidant defense system can be caused by increased production of free radicals and/or a decreased antioxidant capacity in diseased individuals [26].

To the best of our knowledge, this is the first study that compares the salivary antioxidant profile in periodontitis patients with SSc and systemically healthy periodontitis patients. Despite accumulating evidence that oxidative stress plays a major role in the pathogenesis of systemic sclerosis, there are few available studies that analyze the salivary antioxidant status of SSc patients [27, 28]. Furthermore, none of these studies had included a complete periodontal evaluation of systemic sclerosis patients. Given the high prevalence of periodontitis in patients with systemic sclerosis, it is reasonable to assume that the data obtained in this way might be confounded by concurrent periodontal disease.

According to the results of the present study, patients with systemic sclerosis presented a specific periodontal clinical profile which was marked by higher clinical attachment loss and gingival recession values. Nevertheless, periodontal probing depth and gingival index values were not significantly different in the SSc group compared to systemically healthy controls. According to the new classification of periodontal diseases and conditions based on etiopathogenesis, systemic sclerosis (scleroderma) was assigned into the group of systemic diseases that can have an impact on periodontal tissue destruction [29]. However, there are no precise recommendations for when systemic sclerosis should shift the grade score to a higher value independently of the primary criterion represented by the rate of progression [16]. Most of the recent studies showed a higher frequency of periodontitis in SSc patients [30–32]. Pischon et al. found periodontitis in more than 90% of SSc patients associated with high CAL and low gingival inflammation [32], which is in concordance with our results. Higher CAL in SSc patients even after additional adjustments for plaque accumulation was found suggesting multifactorial etiology of periodontal disease in SSc. Similar findings regarding the high prevalence of periodontal disease with a distinct periodontal profile were observed by Gomes da Silva et al. [33].

Even though increased periodontal destruction was detected in the SSc group, PPD values were not significantly different between the groups in our study. This finding may be explained with periodontal microvascular alteration and chronic tissue fibrosis due to limited collagen turnover that might be responsible for increased periodontal destruction with suppressed inflammatory clinical signs [32]. Additionally, tissue fibrosis involving particularly mucosal frena may promote gingival recession [34] and may potentially explain our results.

Significantly higher PI was observed in SSc participants when compared to controls. This finding can be influenced by xerostomia, diminished mouth opening, and impaired manual dexterity in SSc patients [35]. Additionally, a higher number of decayed and filled teeth found in these individuals represent predisposing oral factors that increase the local accumulation of dental plaque [36].

In an effort to better understand the pathogenesis of systemic sclerosis and periodontitis, the present study was designed to determine the levels of antioxidants in unstimulated saliva from periodontitis patients with and without systemic sclerosis. Antioxidative capacity can be determined both in saliva and GCF. Despite the fact that the submandibular and sublingual glands donate the most to the unstimulated saliva, the contribution of GCF is not negligible particularly during gingival inflammation [37]. Although unstimulated saliva has a lower concentration of antioxidants than GCF, several studies show compatibility between the two oral fluid findings, suggesting practical values of using saliva for antioxidant assessment [38–40]. Recent studies revealed the reduced antioxidant capacity of saliva in systemically healthy periodontitis patients [41–43]. Chapple et al. demonstrated the renewal of antioxidant capacity after successful nonsurgical therapy of periodontitis suggesting that gingival inflammation may be the cause of local antioxidant perturbation in patients with chronic periodontitis [42].

The different behavior of antioxidants in unstimulated saliva found in the present study may be explained by the different origins and functions of those antioxidants. SOD and GPX, secreted by the salivary glands, are functioning as part of a preventive antioxidant defense system which inhibits the formation of ROS. GPX and SOD are identified as indirect biomarkers of oxidative stress as there is usually a compensatory increase in concentration as a response to the increasing level of H_2_O_2_ [39]. Similar results regarding a significant increase in the level of GPX in unstimulated saliva in SSc patients compared to healthy controls were found in a study conducted by Zalewska et al. [27]. An increased antioxidative capacity of unstimulated saliva indicates sufficient protection against an increased level of H_2_O_2_ in SSc patients. On the other hand, plasma-born uric acid acts as a radical-scavenging antioxidant which scavenges free radicals to obstruct chain reaction [44]. Knowing the fact that saliva composition mainly depends on plasma composition as well as patients with SSc have an increased concentration of uric acid in plasma [45], it is reasonable to expect elevated levels of UA in SSc individuals. A possible explanation for our results might be a microvascular alteration, particularly defective vascular permeability, previously demonstrated at the early stages of disease [46]. Also, increased level of locally produced free radicals may exhaust local antioxidative capacities leading to decreased UA saliva concentration.

Analysis of the correlations between antioxidants and clinical rheumatological parameters in the SSc group demonstrated two significant correlations (GPX/DAS and SOD/MRSS). Severe inflammation and fibrosis of salivary glands presented in an advanced form of SSc could be a possible cause for these findings considering that most salivary GPX and SOD are locally synthesized in salivary glands as a compensatory reaction to inflammatory stimulation. Since higher MRSS also coincides with more serious internal organ involvement in the diffuse type of SSc [47], it may be assumed that salivary glands are also affected which may potentially explain our results.

The major limitation of our study is the small number of participants. Likewise, it should be kept in mind that all subjects in the SSc group are under immunosuppressive regiments based on corticosteroids, cytotoxic drugs, and calcium channel blockers which may contribute to gingival inflammation and oxidoreductive balance in SSc patients [48]. It is well known that Sjögren's syndrome independently may have an impact on the level of antioxidants in saliva [49]. In the present study, however, we did not perform routine histologic analysis of labial salivary gland biopsies to establish a diagnosis of systemic sclerosis-associated Sjögren's syndrome due to ethical reasons. Since a small number of patients were enrolled in the study, we were not able to subdivide study groups based on salivary flow rate reduction. The other study limitation is the selection of controls in the study. The patients in the study groups were matched regarding the demographic characteristics. It should be emphasized that matching cases and controls according to the stage and grade of periodontitis was challenging due to the fact that SSc is a risk factor for periodontal disease. Even though the patients in the study groups were almost matched regarding the periodontal status (85% of controls exhibited stage III and grade B periodontitis, and all the SSc patients were stage III and grade B periodontitis), future case-control studies on a similar topic are recommended to achieve this level of matching.

Within the limitations of our study, we can conclude that patients with systemic sclerosis presented higher CAL and GR than systemically healthy periodontitis patients. Higher antioxidant perturbations in unstimulated saliva of SSc patients with periodontitis compared to controls were detected. Further research based on a larger population is required to overcome the major limitation of this study and to elucidate the correlation of salivary antioxidants and periodontal status in SSc periodontitis patients.

## Figures and Tables

**Table 1:** Disease characteristics of individuals with systemic sclerosis.

Demographics	SSc group (*n* = 20)
Clinical form (diffuse: limited)	16 : 4
Disease duration (years ± sD)	5.22 (±0.62)
Disease severity and activity (mean ± sD)	
Modified Rodnan's skin score	7.39 (±4.1)
Disease activity score	2.56 (±1.33)
Systemic involvement (no. of patients, %)	
Arthritis	5 (20)
Pulmonary fibrosis	7 (35)
Renal crisis	4 (20)
Esophageal involvement	4 (20)
Heart involvement	5 (25)
Autoantibodies (%)	20 (100)
Antinuclear (ANA)	7 (35)
Antitopoisomerase I (anti-SCL-70)	16 (80)
Anticentromere (ACA)	7 (35)
Treatment (no. of patients, %)	
Corticosteroids	8 (40)
Immunosuppressive drugs	12 (60)
Calcium channel blockers	9 (45)
ACE inhibitors	8 (40)
Antimalarics	1 (5)

**Table 2:** Dental and periodontal findings and antioxidant concentrations among the SSc and P groups.

Variables	SSc group (*n* = 20) $\overset{®}{\text{X}}\pm \text{SE}$ (med, IQR)	P group (*n* = 20) $\overset{®}{\text{X}}\pm \text{SE}$ (med, IQR)	*p* value^a^
IID (mm)	31.3 ± 2.3 (26.4; 35.2)	44.3 ± 1.2 (41.6; 46.1)	≤0.001^∗^
Number of teeth	13.61 ± 0.63 (12.5; 2)	18.43 ± 0.74 (17.1; 2.1)	≤0.001^∗^
DMFT	18.64 ± 6.11 (17.5; 1.1)	12.32 ± 6.85 (11.3; 2.0)	≤0.001^∗^
CAL (mm)	4.8 ± 0.21 (4.85; 1.23)	3.18 ± 0.17 (3.20; 1.33)	≤0.001^∗^
PPD (mm)	3.17 ± 0.26 (3.05; 1.55)	3.65 ± 0.09 (3.67; 0.48)	0.123
GR (mm)	1.66 ± 0.90 (0.00; 3.50)	0.46 ± 0.54 (0.42; 1.50)	≤0.001^∗^
GI	1.09 ± 0.21 (0.90; 1.75)	1.49 ± 0.07 (1.5; 0.4)	0.107
PI	2.56 ± 0.24 (3; 0.02)	1.28 ± 0.06 (1.3; 0.4)	≤0.001^∗^
SOD (IU/L)	0.30 ± 0.17 (0.28; 0.68)	0.48 ± 0.06 (0.5; 0.4)	0.021^∗^
GPX (IU/L)	322.11 ± 29.35 (315; 103.0)	1839.427 ± 30.63 (1833.6; 234.6)	≤0.001^∗^
UA (*μ*M)	257.89 ± 36.02 (195; 249)	154.20 ± 5.34 (154; 35)	0.083

Abbreviation: IID: interincisal distance; DMFT: decayed, missing, filled teeth index; CAL: clinical attachment loss; PPD: periodontal pocket depth; GR: gingival recession; GI: gingival index; PI: plaque index; SOD: superoxide dismutase; GRX: glutathione peroxidase: UA: uric acid; sd: standard deviation; X̄: mean; SE: standard error; Med: median; IQR: interquartile range. ^a^Variables were compared between two groups using the Mann–Whitney *U* test. ^∗^Statistical significance (*p* ≤ 0.05).

**Table 3:** Correlation among antioxidants and periodontal clinical parameters in the SSc group.

		PI	GI	PPD	CAL	GR
GPX	*R*	0.485	0.231	0.166	-0.016	0.200
	*p*	0.081	0.355	0.509	0.951	0.938

SOD	*R*	0.113	0.319	0.204	-0.032	0.381
	*p*	0.654	0.197	0.416	0.899	0.118

UA	*R*	0.351	0.423	0.122	0.101	-0.005
	*p*	0.154	0.081	0.631	0.691	0.985

PI: plaque index; GI: gingival index; PPD: periodontal pocket depth; CAL: clinical attachment loss; GR: gingival recession; GPX: glutathione peroxidase; SOD: superoxide dismutase; UA: uric acid.

**Table 4:** Correlation among antioxidants and periodontal clinical parameters in the P group.

		PI	GI	PPD	CAL	GR
GPX	*R*	-0.111	0.099	0.143	0.241	-0.207
	*p*	0.693	0.725	0.611	0.387	0.459

SOD	*R*	0.006	-0.011	-0.101	-0.134	0.092
	*p*	0.982	0.968	0.720	0.635	0.743

UA	*R*	0.007	0.042	0.227	0.118	0.010
	*p*	0.979	0.881	0.417	0.675	0.971

PI: plaque index; GI: gingival index; PPD: periodontal pocket depth; CAL: clinical attachment loss; GR: gingival recession; GPX: glutathione peroxidase; SOD: superoxide dismutase; UA: uric acid.

**Table 5:** Correlation between clinical rheumatological variables and level of salivary antioxidants in the SSc group.

		GPX	SOD	UA
Disease duration	*R*	0.061	-0.237	0.368
	*p*	0.810	0.345	0.133

Modified Rodnan's skin score	*R*	-0.203	0.607	-0.105
	*p*	0.419	0.008^∗^	0.679

Disease activity score	*R*	-0.607	0.324	-0.178
	*p*	0.008^∗^	0.189	0.481

GPX: glutathione peroxidase; SOD: superoxide dismutase; UA: uric acid. ^∗^Statistical significance R—Pearson's correlation coefficient.

## Data Availability

The data used to support the findings of this study have been deposited in the ClinicalTrials.gov (https://clinicaltrials.gov/ct2/home), ID number: NCT05215431.
